# AI-driven infrared spectroscopy in salivary oral leukoplakia

**DOI:** 10.1007/s00784-026-07246-3

**Published:** 2026-11-05

**Authors:** Sara Maria Santos Dias da Silva, Jaqueline Maria Brandão Rizzato, Ana Carolina Yumi Taromaru, Yuri de Lima Medeiros, Fábio Abreu Alves, Thiago Martini Pereira, Camila de Barros Gallo, Fabianne Soares Lima, Luis Felipe das Chagas e Silva de Carvalho

**Affiliations:** 1 University of Taubaté – UNITAU – Taubaté, Rua dos Operários, 09, Centro, Taubaté, São Paulo, SP 1202-340 Brazil; 2 Department of Stomatology, School of Dentistry, University of São Paulo – USP – São Paulo, São Paulo, Brazil; 3 Department of Stomatology, A.C. Camargo Cancer Center, São Paulo, Brazil; 4 Federal University of São Paulo, UNIFESP – São José dos Campos, São José dos Campos, São Paulo Brazil

**Keywords:** Saliva, FTIR spectroscopy, Machine learning, Leukoplakia, Oral potentially malignant disorders

## Abstract

**Objectives:**

Oral potentially malignant disorders (OPMDs), particularly leukoplakia, are strongly linked to an increased risk of oral squamous cell carcinoma. Saliva has gained attention as a diagnostic biofluid due to its simple, non-invasive collection and complex biochemical composition. This study aimed to investigate the application of Fourier-transform infrared (FTIR) spectroscopy combined with machine learning to identify salivary biochemical changes.

**Materials and methods:**

Saliva samples were collected from healthy control participants (*n* = 22), participants with oral leukoplakia (*n* = 22). Spectral data were acquired by ATR-FTIR, pre-processed with derivative analysis, normalization, and baseline correction, then analyzed using PCA and classified with Extra Trees, Random Forest, and XGBoost algorithms.

**Results:**

The analysis revealed consistent biochemical differences within the 900–1800 cm⁻¹ region, mainly associated with proteins (Amide I and II), nucleic acids, and collagen. PCA revealed partial but relevant group separation, while machine learning models, especially Extra Trees and Random Forest, the MLP classifier achieved an AUC of 0.992 for initial dysplasia and 0.974 for severe dysplasia versus controls, indicating promising classification perfomance.

**Conclusion:**

These findings support FTIR spectroscopy integrated with machine learning, as a powerful, non-invasive approach for salivary profiling in leukoplakia. This optical biopsy method shows promise as an auxiliary diagnostic tool for early detection and monitoring of OPMDs, enhancing preventive strategies in oral oncology.

## Introduction

Saliva is a biocompound secreted by the salivary glands. These glands are divided into two groups: major and minor glands. Major glands are located outside the oral cavity and are represented by three pairs, including the parotid, sublingual and submandibular glands. Minor glands are represented by small secretory units located inside the oral cavity [1, 2]. Saliva is composed primarily of water (approximately 99%), with the remaining fraction containing, in smaller quantities, several other molecules can be found, such as sodium, potassium, chlorine, calcium, bicarbonate, magnesium ions and other substances such as amino acids, triglycerides, nitrogen compounds such as urea and ammonia, in addition to more than 3,000 proteins, the most prevalent being aPRP, bPRP, mucins and amylase [3, 4].

Salivary secretion also performs essential functions, including: in digestion, where it moistens food to facilitate digestion, in addition to playing a minimal role in the digestion of carbohydrates through amylase; it aids in taste, since it solubilizes food so that it can be perceived by taste receptors; it protects the oral cavity from bacteria, through salivary proteins: lysozyme, lactoferrin and lactoperoxidase, which have the ability to lyse bacteria or prevent them from developing; it maintains a neutral pH in the oral cavity [2–4]. In addition, it has been standing out to diagnose several diseases [2]. This is due to its ease of collection, storage and because it is a non-invasive method that does not pose risks to health professionals and patients [4, 5].

Changes in oral health can lead to changes in salivary glands, such as their genetic expressions and, consequently, their proteins, which can generate biomarkers for various conditions [6]. In this article, we highlight some changes related to potentially cancerous lesions, mainly in relation to leukoplakia and erythroplakia. These types of lesions, in addition to generating genetic changes, can also lead to the production of cytokines, such as IL-6, IL-8 and TNF. The more advanced the lesions, the greater the amounts of these markers will be found, due to the increase in inflammation in these areas [7].

Leukoplakia and erythroplakia, together with oral submucous fibrosis and proliferative verrucous leukoplakia, are considered the main OPMD in relation to the chance of malignancy, although all OPMDs are lesions that generate an increased risk for the most prevalent oral cancer - squamous cell carcinoma [6–8].

For OPMDs to be diagnosed, there is first a clinical suspicion that is determined by the patient’s history, signs and symptoms and changes in the mucosa [7, 8]. In the case of leukoplakia, white plaques appear that cannot be removed, and in the case of erythroplakia, they are reddish and flat lesions. Thus, these lesions are collected by means of a biopsy, to undergo histopathological examination and confirm the suspicion [6–8]. This diagnosis is made based on the classification of the degree of cellular differentiation, which can be: mild, moderate or severe epithelial dysplasia, with dysplasia being when cellular growth presents an abnormal pattern, such as in the amount of cytoplasm, size and quantity of nucleus or irregular cell shape [7–10].

Thus, techniques that enable rapid diagnosis significantly to favor the treatment and prognosis of patients with possible pre-cancerous lesions, systemic diseases or rapidly evolving diseases, and are therefore of extreme importance to society [9, 10]. Thus, the faster the return of the requested tests, the better the chances of a favorable clinical outcome. In contrast to tests already consolidated in clinical practice, such as blood counts and biopsies, vibrational spectroscopy has the advantage of providing biochemical information on samples in real time and without the need for prior preparation [11].

Vibrational spectroscopy (VS) is a branch of spectroscopy that analyzes molecular vibrations, which are specific characteristics of each chemical structure. In polyatomic molecules, these vibrations generate a unique spectral “fingerprint” [11]. The resulting spectrum can be used to characterize the molecular structure or identify changes associated with changes in the local environment or external factors, such as radiation or chemical agents [12]. Among the VS techniques, Fourier transform infrared spectroscopy (FTIR) has established itself as a valuable tool in biophysics for the analysis of proteins, lipids, carbohydrates, and nucleic acids [11–13].

In this study, we analyze the use of FTIR spectroscopy for the spectral characterization of saliva from patients with oral leukoplakia, with the aim of creating a database for comparison. From this approach, we seek to identify characteristic biochemical patterns of saliva, which can be used as comparison parameters with samples from individuals with pathological processes, such as inflammatory lesions, benign and malignant neoplasms. In this way, we intend to contribute to the development of a non-invasive, fast and accurate auxiliary diagnostic technique, through an “optical biopsy” approach based on FTIR spectroscopy.

## Methodology

This work is funded by FAPESP under process number 2023/01749-7 and was approved by the Research Ethics Committee of the University of Taubaté (São Paulo, SP, Brazil), under number 19436919.7.0000.5501. All volunteers were over 18 years of age, regardless of gender, and before the saliva samples were collected, these volunteers signed the Free and Informed Consent Form (FICF), and the saliva collection was performed at the Department of Dentistry of the University of Taubaté and Department of Stomatology, School of Dentistry, University of São Paulo – USP – São Paulo, São Paulo.

Infrared spectra were acquired using Alpha II (Bruker Optics, Germany), with spectral acquisition managed using OPUS software (Bruker). The spectral data processing and analysis were performed in Python 3.10 within the Anaconda distribution, using custom-written routines available at (https://github.com/tmpereira/spc*).* Prior to multivariate analysis, a preprocessing pipeline was applied to remove spectral variations not associated with the biochemical differences among samples.

Spectral preprocessing consisted of three sequential steps: (i), the second derivative was computed using the Savitzky-Golay (SG) algorithm with an 11-point window and a second-order polynomial fit. The second derivative eliminates all baseline distortions up to second order—including constant offsets, linear tilts, and parabolic curvature—arising from the optical heterogeneity of the sample, as well as from detector drift and non-specific absorption. The choice of an 11-point window represents a compromise between spectral resolution (narrower windows preserve peak sharpness) and noise amplification (wider windows provide stronger smoothing); a second-order polynomial was selected because it adequately models the local curvature of mid-infrared bands without introducing spectral distortion; (ii), spectral analysis was restricted to the fingerprint region between 900 and 1800 cm^− 1^. This region contains the most diagnostically informative vibrational modes of biological macromolecules, including C–O, C–N, and C–C stretching, P–O stretching of nucleic acid phosphodiester backbones, amide I (~ 1650 cm^− 1^) and amide II (~ 1550 cm^− 1^) bands of proteins, and carbohydrate ring deformations, among others and (iii), each preprocessed spectrum was subjected to vector normalization (also known as unit-vector or Euclidean normalization), in which the absorbance value at every wavenumber is divided by the Euclidean norm (root-sum-of-squares) of the entire spectral vector. This procedure scales all spectra to unit length I n multidimensional spectral space, thereby removing multiplicative intensity differences due to variations in sample concentration, effective path length through the ATR crystal.

Following preprocessing, Principal Component Analysis (PCA) was performed on the resulting dataset. PCA is an unsupervised exploratory technique that transforms the data into a new set of orthogonal variables, called Principal Components (PCs). This transformation reduces data dimensionality by concentrating the maximum variance into the first few PCs, thereby removing multicollinearity inherent in the original spectral variables.

A total of 20 samples per group (20 healthy vs. 20 leukoplakia) were analyzed, resulting in 40 samples; each biological sample was analyzed in triplicate, resulting in a total of 120 spectra. The leukoplakia cohort comprised 20 cases with available demographic and histopathological information, including 14 females (70.0%) and 6 males (30.0%). Histopathological findings included severe dysplasia in 9 cases (45.0%), initial dysplasia in 11 cases (55.0%). These histopathological categories were used to characterize the study population and inform the exploratory subgroup analyses.

### Inclusion and exclusion criteria

The patients chosen for the development of this project followed the following criteria: Participants over 18 years of age, without distinction of sex, no pre-existing diseases, no use of legal or illegal drugs, and any type of cigarette use, whether straw, electronic, tobacco, among others. All patients included in this study were subjected to a comprehensive intraoral clinical examination and cervical lymph node palpation to ensure the absence of any oral cavity disorders prior to sample collection. Therefore, volunteers who did not fit these criteria were excluded. The volunteers in this study were instructed to clean their oral cavity with filtered water, suitable for consumption, before collecting saliva. Also, as an exclusion criterion after collection, a test was performed on each patient for fasting blood glucose, normal blood glucose, HDL, LDL, C-reactive protein and glycated hemoglobin. If any of these parameters were altered, the patient was excluded.

### Collect and processing

Unstimulated whole saliva samples were collected by a single researcher using the spit/expectoration method. Participants were instructed to rinse their mouths with water for 30 s and remain for one minute without swallowing and then spit out all accumulated saliva (approximately 5 ml) into a sterile millimeter tube for collection. The samples were subsequently stored in an eppendorf tube, after which the samples were kept in a freezer frozen at -80 °C, thawed and kept in the freezer at -20 °C and later analyzed in the spectrometer, to be analyzed by vibrational spectroscopy using the ATR FTIR technique.

The spectrometer used was the Alpha II (Bruker Optics) ATR FTIR Spectrometer, which has a diamond crystal with temperature regulation in 37°c for spectral data acquisition. In the equipment, the samples were inserted without additives under the crystal with the aid of a pipette calibrated at 1 µl to obtain the spectra. After being placed on the crystal, the samples were allowed to dry completely to obtain better spectra. The drying time varied from 2 to 7 min with an average time of 5 min. The analysis was performed with 32 scans and background to obtain the average of the spectra with a resolution of 4 cm-¹ and removal of possible interferences. All spectra were obtained in triplicate, following a previous study [14]. After each analysis, the samples were removed from the crystal with the aid of absorbent paper and cleaned with 92.8% alcohol to completely remove the samples from the crystal, avoiding contamination between samples.

The spectrum was pre-processed, which consisted of FTIR spectrum smoothing, vector normalization and baseline and background removal using the second derivative of the spectrum, to make them suitable for comparison in the analysis step. Artifacts inherent to spectral acquisition, including instrumental noise, baseline drift, and variability due to sample thickness and surface interaction, were minimized through the combined application of smoothing, second derivative transformation, baseline correction, and normalization. These procedures ensured preservation of relevant spectral information without compromising signal integrity.

### Data analysis

After the analysis performed by the spectrometer, the raw graph will be compiled using mathematical tools that assist in the results obtained, thus having the purpose of normalization through a vibrational mode or vector normalization. In this context, to perform the baseline correction, normalization, smoothing and study of the second derivative of the spectra, the Python 3.10 (Anaconda distribution). For effective analysis of the spectra with the purpose of promoting the diagnosis of the studied samples through the biochemical characteristics evidenced in this study, the spectra were processed and analyzed in stages:

There are some artifacts that were included in the spectra during their acquisition, such as noise, and thus in the smoothing stage they could be minimized without compromising the collected signal. Therefore, the methodology used to remove fluorescence was Baseline correction.

Small differences in intensity between the spectra could also occur as a result of experimental factors, such as minimal variations in intensity due to the type of surface of the samples. Therefore, the spectra were standardized through normalization, smoothing, and baseline correction, and thus analyzed so that any differences that were evidenced were only biochemical characteristics.

**Fig. 1:**
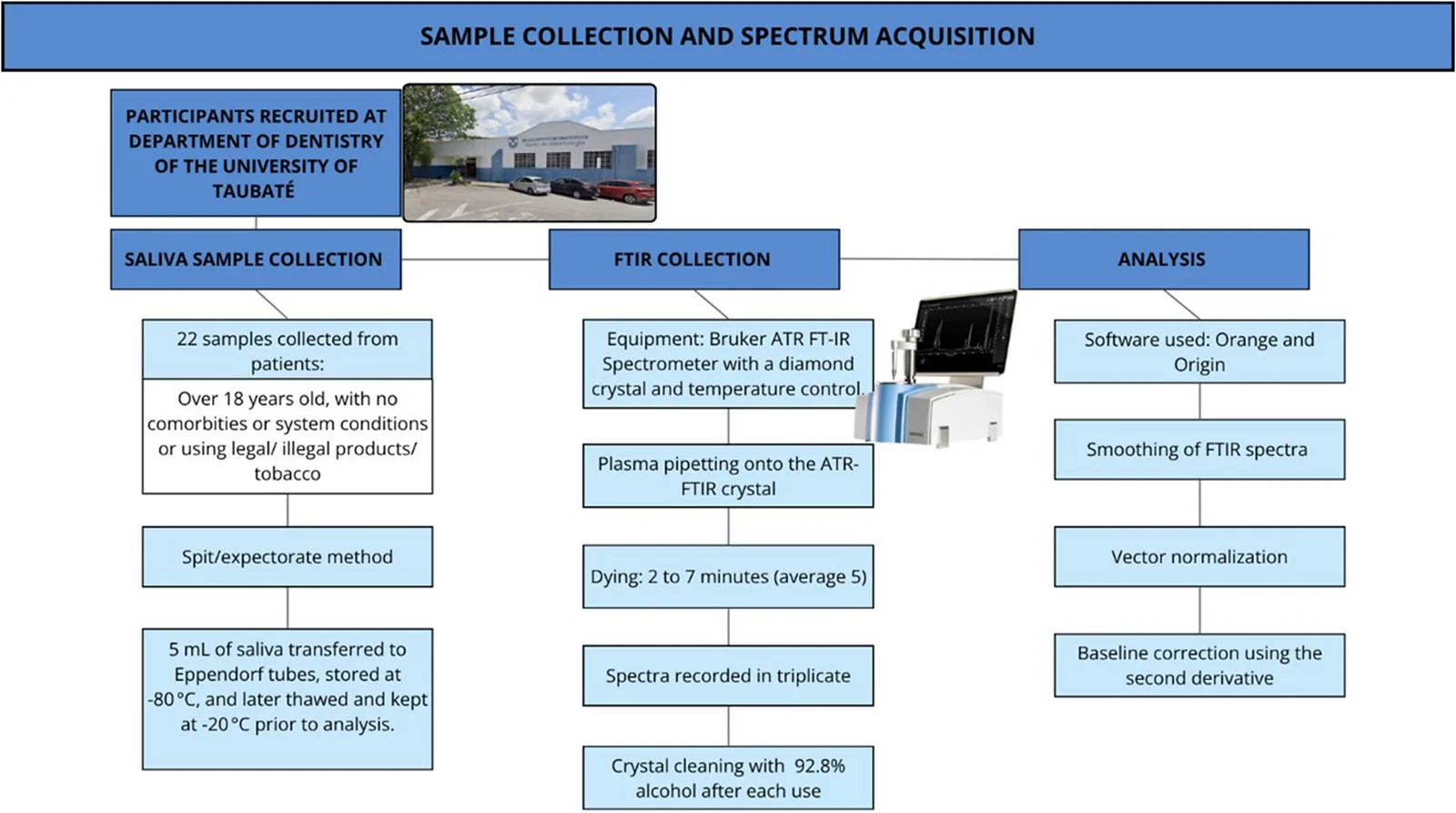
Schematic representation of the experimental workflow, from saliva sample collection to FTIR spectral acquisition. The flowchart details the sequential steps of sample collection, storage, preparation, and infrared spectral acquisition using an ATR-FTIR system

The workflow from saliva sample collection to spectral acquisition is illustrated in Fig. 1, summarizing the sequential steps of sample preparation, storage, and FTIR analysis:

## Results

**Fig. 2:**
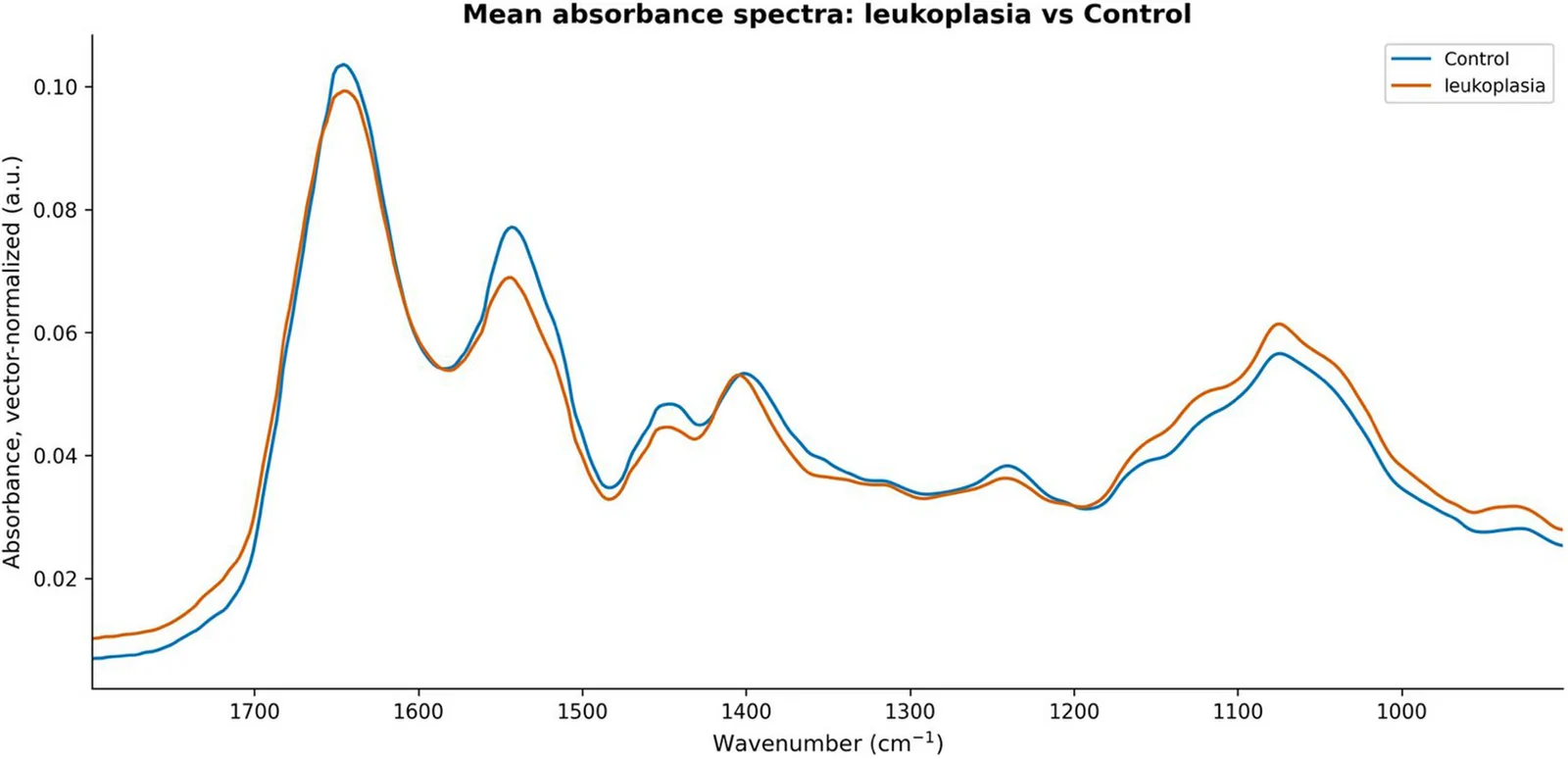
Mean FTIR absorbance spectra of saliva sample from patients with oral leukoplakia and control without oral lesions in the 900–1800 cm^-1^ spectral region

The mean FTIR absorbance spectra of saliva samples from patients with oral leukoplakia and control without oral lesions (Fig. 2) showed similar overall spectra profiles, with differences in the relative intensities of several absorption bands. Both groups exhibited a prominent band around 1645 cm-1 and a second feature near 1545 cm-1. The control group displayed a higher mean absorbance in these regions, particularly around 1545 cm^-1^. In contrast, the leukoplakia group showed higher mean absorbance in the approximately 1150–1050 cm^-1^ region. The spectra also showed closely overlapping profiles in several other regions, including around 1400 cm^-1^ and 1200 cm^-1^. These observations indicate subtle differences in the mean spectral profiles between the groups, alongside substantial overall similarity.

**Fig. 3:**
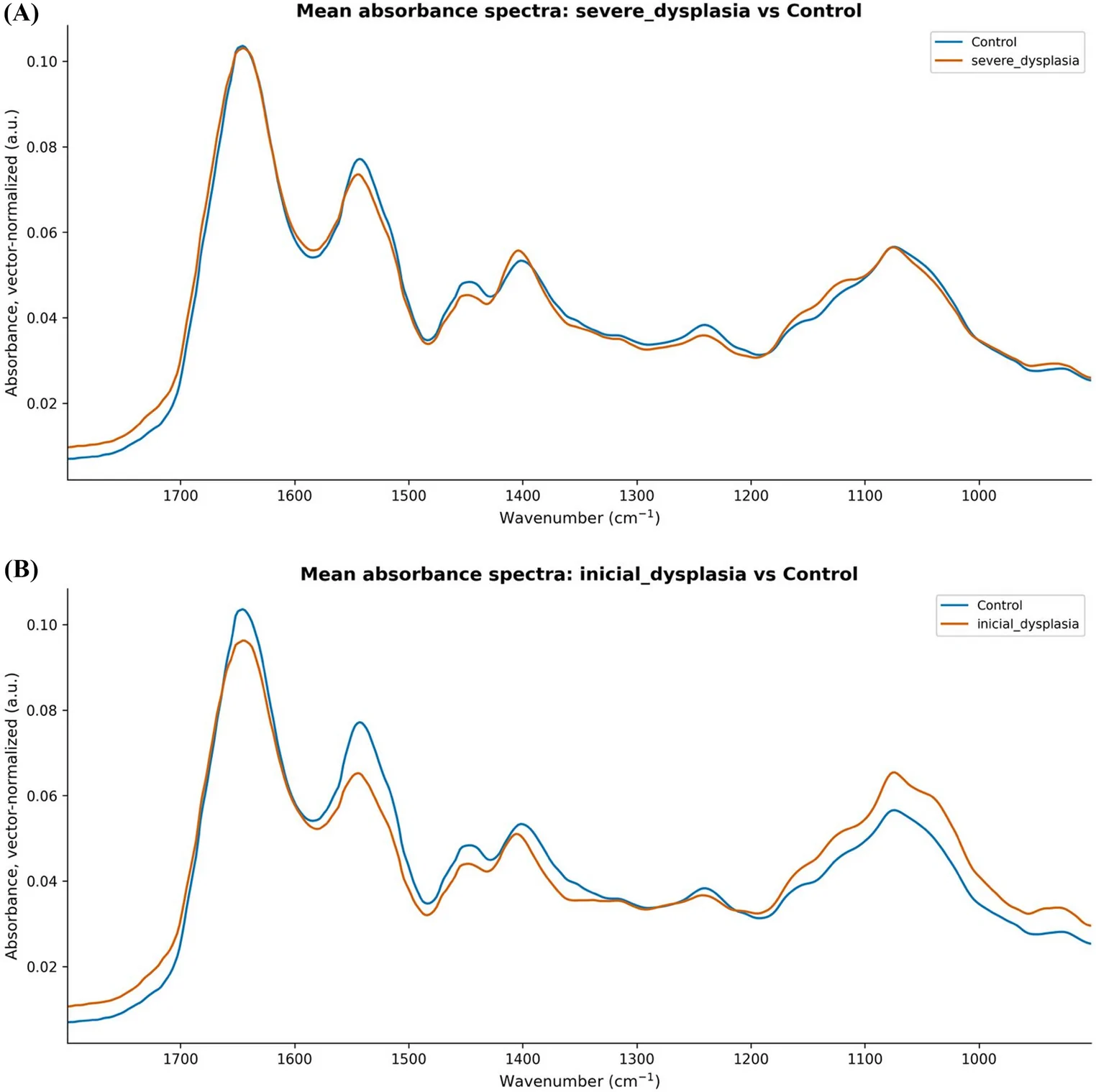
Comparison of mean FTiR absorbance spectra of saliva sample from controls and patients with oral leukoplakia according to histopathological subgroup. **A** Severe dysplasia group versus controls. **B** Initial dysplasia group versus controls. Spectra are shown in the fingerprint region (900–1800 cm-1). Differences in relative absorbance intensities are observed across selected spectral regions, while substantial overlap between the groups remains

The comparison of mean vector-normalized FTIR spectra revealed differences in the spectral profiles of the control group and the two histopathological subgroups (Fig. 3). In the severe dysplasia group, the overall spectra profile closely resembles that of the controls, with the most prominent differences observed around 1545 cm^-1^, where the control group exhibited higher absorbance, and in the region between approximately 1150 and 1050 cm^-1^, where the profiles showed small variations in relative intensity. The band around 1645 cm^-1^ was similar in both groups.

In contrast, the initial dysplasia group exhibited more noticeable differences from the controls in several spectral regions. Lower mean absorbance was observed around 1645 and 1545 cm^-1^, whereas higher absorbance was evident between approximately 1150 and 1050 cm^-1^. Differences were also visible around 1400 cm^-1^, although the profiles remained broadly similar in this region. Overall, the initial dysplasia spectra showed more pronounced differences in relative band intensities than those observed in the severe dysplasia group. These findings describe visual differences between mean spectra.

Characterization table (Table 1) of the results obtained:

**Table 1:** Main spectral peaks identified in the samples and their corresponding biochemical assignments

Bands (cm^-1^)	Wave definition	Vibrational modes	Refs.
~ 1645	Amide I	Mainly C = O stretching of peptide bonds; sensitive to protein secondary structure.	[12, 19, 22]
~ 1545	Amide II	Coupled N-H bending and C-N stretching in proteins.	[12, 19, 22]
~ 1450	Protein/lipid-associated region	CH_2_ and CH_3_ bending and deformation vibrations.	[19, 22]
~ 1400	Carboxylate/protein-associated region	Symmetric stretching of COO^-^ groups and deformation of CH_3_ groups.	[19, 22]
~ 1240	Amide III/phosphate-associated region	C-N stretching and N-H bending; possible contribution from asymmetric PO_2_^-^ stretching.	[19, 22]
~ 1150	Protein/carbohydrate-associated region	C-O stretching and vibrations involving C-O-C groups.	[19, 22]
~ 1100 − 1017	Phosphate/carbohydrate-associated region	P-O and PO_2_^-^ vibrations, with possible contributions in carbohydrate structures.	[19, 22]
~ 1000 − 950	Fingerprint region	Overlapping C-O and C-C vibrations, with possible contributions from other biomolecular constituents.	[19, 22]

**Fig. 4:**
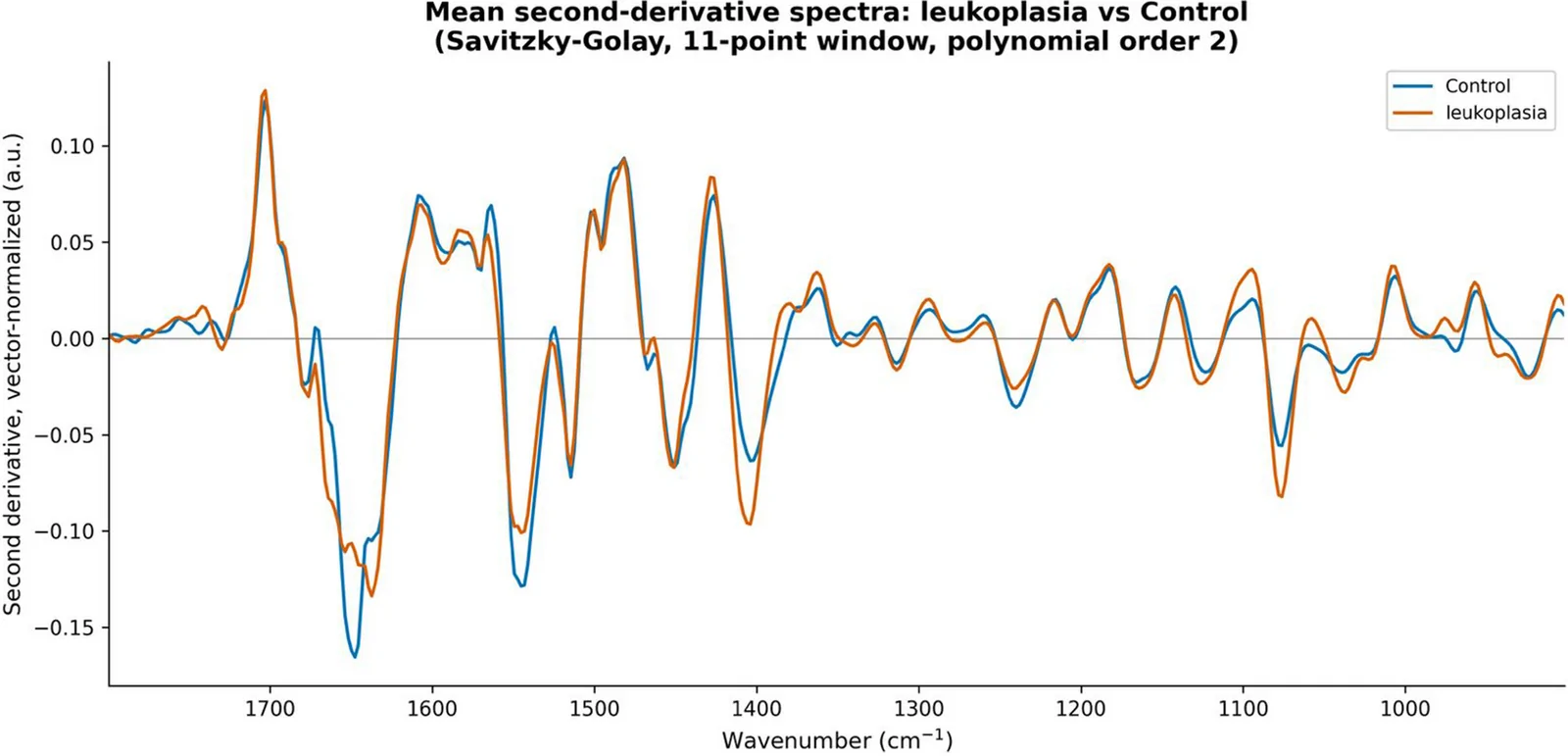
Mean second-derivative FTIR spectra of saliva samples from the control and oral leukoplakia groups in the fingerprint region

Savitzky–Golay second-derivative spectra were examined to compare changes in band shape and local spectral features after reducing the influence of broad baseline variations. The processing used an 11-point window and a second-order polynomial. The second-derivative profiles of the control and leukoplakia groups displayed similar patterns of positive and negative features across the analysed fingerprint region. However, differences in the magnitude of selected features were apparent, particularly around 1650–1630, 1560–1540, 1450–1400, and approximately 1080 cm⁻¹. Similar local variations were observed in the initial and severe dysplasia comparisons. In the initial dysplasia group, differences around 1550 cm⁻¹ and 1080 cm⁻¹ were particularly noticeable, while the severe dysplasia comparison showed differences in the magnitude of features around 1545 and 1400 cm⁻¹ (Fig. 4).

Despite these local differences, the second-derivative spectra also showed considerable overlap between the groups. Thus, the visual comparisons indicate differences in spectral shape and relative band intensity rather than complete spectral separation. The second-derivative plots are descriptive and do not, by themselves, establish statistical significance or identify specific molecular changes.

The mean spectra with shaded regions representing ± 1 standard deviation showed variation in spectral dispersion across the fingerprint region. In the initial dysplasia comparison, the variability was particularly apparent around the main bands near 1650 and 1545 cm⁻¹ and in the region between approximately 1100 and 1000 cm⁻¹. In the severe dysplasia comparison, variability was also visible around 1650–1630 cm⁻¹ and at selected features between approximately 1500 and 1350 cm⁻¹.

Principal component analysis (PCA) was performed separately for the initial dysplasia and severe dysplasia comparisons with the control group. In the initial dysplasia analysis, the first principal component (PC1) explained 32.20% of the total variance, while the second principal component (PC2) explained 19.41%. Together, these components accounted for 51.62% of the total variance. In the severe dysplasia analysis, PC1 explained 42.21% of the variance and PC2 explained 16.44%, corresponding to a cumulative explained variance of 58.65%. The first three principal components accounted for 69.87% of the variance in this analysis.The partial overlap observed between the groups in the PCA score plots indicates that the salivary biochemical profiles of patients with oral leukoplakia and healthy individuals are not completely distinct. This finding may reflect both the biological heterogeneity of oral leukoplakia and interindividual variability in salivary composition. It should also be considered that PCA is an unsupervised exploratory method that identifies the main sources of variance in the dataset without using diagnostic labels. Consequently, the components accounting for the greatest overall spectral variance are not necessarily those that provide the best discrimination between clinical groups. Although supervised machine learning algorithms can identify subtler multivariate patterns, the PCA overlap reinforces that salivary ATR-FTIR spectroscopy should not currently be considered a stand-alone diagnostic test.

The supervised classification analysis showed differences in performance among the evaluated machine-learning models. The multilayer perceptron (MLP) classifier achieved the highest reported mean accuracy, at 0.967 ± 0.067, and an area under the receiver operating characteristic curve (AUC) of 0.994 ± 0.011. Its mean recall, precision, and F1-score were all 0.967 ± 0.067. The Extra Trees classifier also showed high performance, with an accuracy of 0.950 ± 0.067, an AUC of 0.983 ± 0.022, and an F1-score of 0.953 ± 0.060. The following Table 2 shows the performance of the results that have shown promise for machine learning used to classify or predict the database obtained by analyzing the spectra of pre-cancerous lesions. In this way, metrics (such as AUC, Recall, Kappa, etc.) were used; this is a comparative performance analysis.

**Table 2:** mean ± SD across ten participant-level folds

Model	Accuracy	AUC	Recall	Precision	F1
Random Forest	0.740 ± 0.127	0.845 ± 0.135	0.797 ± 0.159	0.782 ± 0.168	0.771 ± 0.117
Extra Trees	0.729 ± 0.133	0.840 ± 0.153	0.778 ± 0.192	0.773 ± 0.172	0.755 ± 0.133
XGBoost	0.701 ± 0.123	0.820 ± 0.139	0.736 ± 0.201	0.747 ± 0.143	0.723 ± 0.133

**Fig. 5:**
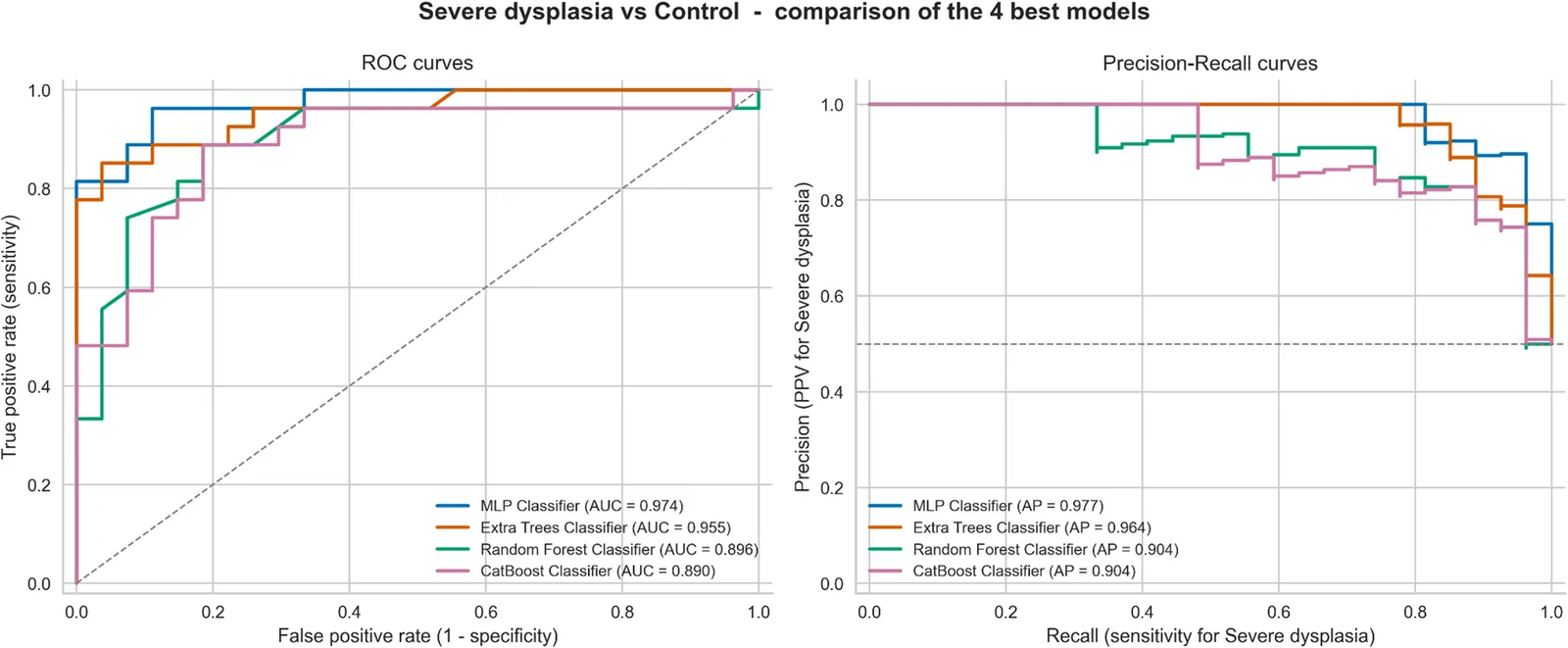
Comparison of the four best-performing machine learning classifiers for distinguishing initial dysplasia from controls

The Random Forest classifier achieved an accuracy of 0.883 ± 0.125 and an AUC of 0.958 ± 0.057, while CatBoost achieved an accuracy of 0.917 ± 0.129 and an AUC of 0.955 ± 0.059. Performance varied across the remaining models. For example, the linear support vector machine achieved an accuracy of 0.750 ± 0.139, whereas the radial-basis-function support vector machine achieved an accuracy of 0.550 ± 0.041. Overall, the reported results suggest that the initial dysplasia-versus-control comparison was classified more effectively by the MLP and Extra Trees models than by several of the other evaluated algorithms. These findings are exploratory and should be interpreted in the context of the sample size, the validation strategy, and the absence of an independent external test cohort (Fig. 5).

For the severe dysplasia-versus-control comparison, the MLP classifier achieved the highest reported mean accuracy, at 0.891 ± 0.068, with an AUC of 1.000 ± 0.000. Its mean recall was 0.893 ± 0.137, precision was 0.917 ± 0.105, and F1-score was 0.891 ± 0.066. The Extra Trees classifier achieved an accuracy of 0.835 ± 0.105 and an AUC of 0.967 ± 0.042. Random Forest showed the same reported mean accuracy of 0.835 ± 0.105, with an AUC of 0.903 ± 0.067. CatBoost achieved an accuracy of 0.853 ± 0.108 and an AUC of 0.895 ± 0.060. The remaining classifiers generally showed lower or more variable performance; for example, the radial-basis-function support vector machine achieved an accuracy of 0.504 ± 0.098 and an AUC of 0.731 ± 0.170.

**Fig. 6:**
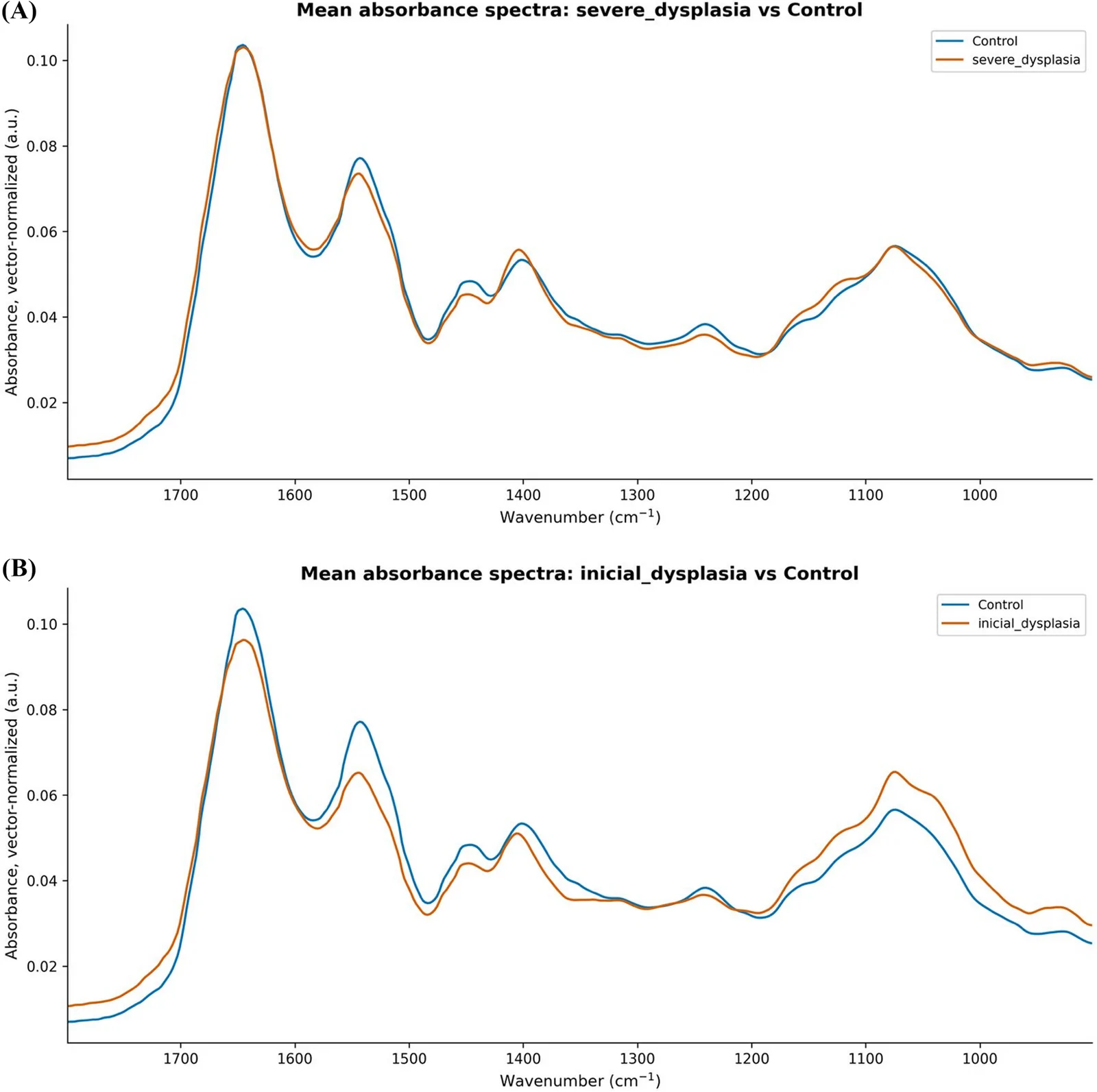
Comparison of the four best-performing machine learning classifiers for distinguishing severe dysplasia from controls

Compared with the initial dysplasia analysis, the severe dysplasia comparison showed lower mean accuracy for the best-performing model, although the reported MLP AUC was higher. The differences between these results should be interpreted cautiously because the comparisons involve different subgroup sizes and potentially different levels of within-group heterogeneity (Fig. 6).

**Fig. 7:**
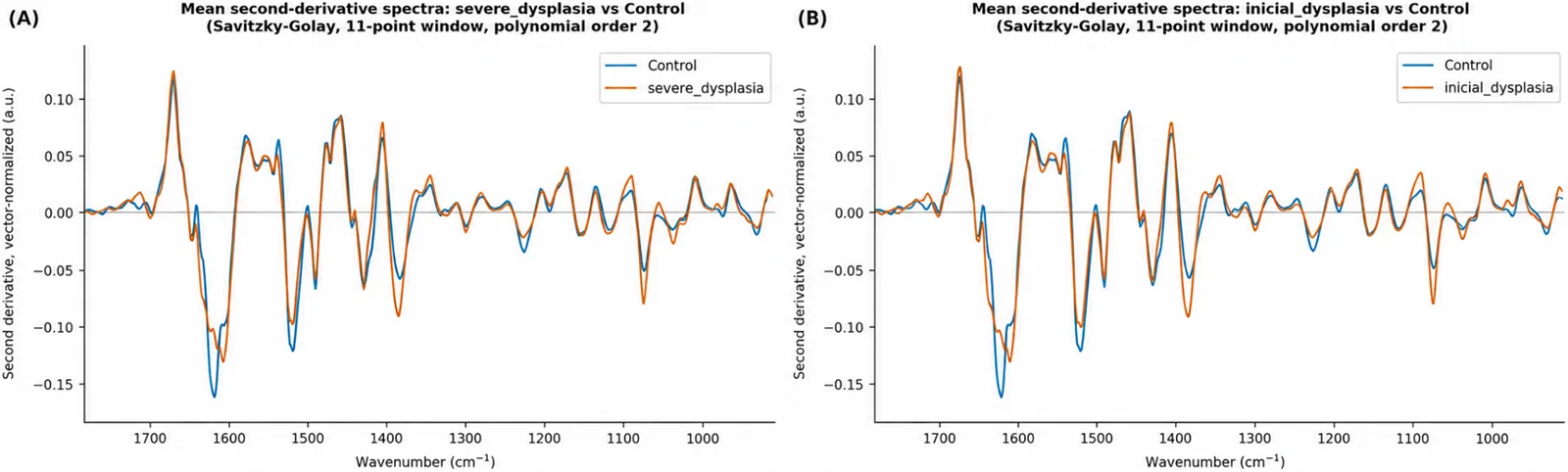
Mean second-derivative FTIR spectra of saliva samples from control groups and patients with (A) severe dysplasia and (B) initial dysplasia

The mean FTIR spectra and their second derivatives revealed differences in relative spectral features between controls and leukoplakia cases, including the initial and severe dysplasia subgroups (Fig. 7). However, substantial overlap remained between the mean profiles. The supervised analyses yielded promising internal classification results, particularly for the MLP and Extra Trees models in the initial dysplasia comparison and for the MLP model in the severe dysplasia comparison. These findings support further investigation of saliva FTIR spectroscopy combined with machine learning as a potential approach for characterising oral leukoplakia-associated spectral patterns. Independent validation in a larger and clinically representative cohort is required before diagnostic performance or clinical applicability can be established.

Despite the promising classification results, the present study demonstrates analytical feasibility rather than clinical validity or utility. Before clinical implementation, the proposed approach must be evaluated in prospective studies involving independent populations and clinically relevant control groups, including patients with inflammatory, traumatic, reactive, and other potentially malignant oral disorders. External validation will also be necessary to determine whether model performance is maintained across different populations, operators, instruments, and clinical settings. At this stage, salivary ATR-FTIR spectroscopy should be regarded as a potential adjunctive screening or risk-stratification approach and not as a replacement for clinical examination, biopsy, or histopathological assessment.

## Discussion

Saliva analysis can facilitate the diagnosis of various pathologies, such as cardiovascular, endocrine, viral, oncological or neurological disorders [15]. This is because during these processes saliva may be altered, either in its composition or properties, such as through the presence of markers or changes in the quantity of materials such as proteins, enzymes, hormones, nucleic acids and other metabolites. In this sense, spectroscopy, when performing salivary analysis, can help in the evaluation of the compounds present in saliva for a possible diagnosis [14, 15].

The relatively small sample size represents an important limitation of the present study. Although the inclusion of 22 patients with oral leukoplakia and 22 healthy individuals allowed an initial investigation of salivary biochemical differences, this cohort may not capture the complete clinical and biological heterogeneity of oral leukoplakia. Moreover, classification metrics obtained from small datasets may be optimistic and particularly sensitive to the composition of the training and testing subsets. Therefore, the reported accuracy and area under the receiver operating characteristic curve should be interpreted as preliminary estimates that require confirmation in larger and independent populations.

In spectroscopy, the main bands represent the concentration of the main macromolecules: lipids, proteins, nucleic acids and carbohydrates [12]. In the research conducted, we can observe differences between the graphs of the two groups in some portions, such as between the peaks between 1400 and 1600 cm^-1^, which may indicate discrepancies in the concentration of proteins, due to metabolic alteration and the presence of protein markers. According to Camisasca et al., in their study that compared saliva from people with leukoplakia and healthy people, there are proteins that increase their concentration during the disease, namely the CK10 fragment and apolipoprotein A1 [16].

The partial overlap observed between the groups in the PCA score plots indicates that the salivary biochemical profiles of patients with oral leukoplakia and healthy individuals are not completely distinct. This finding may reflect both the biological heterogeneity of oral leukoplakia and interindividual variability in salivary composition. It should also be considered that PCA is an unsupervised exploratory method that identifies the main sources of variance in the dataset without using diagnostic labels. Consequently, the components accounting for the greatest overall spectral variance are not necessarily those that provide the best discrimination between clinical groups. Although supervised machine learning algorithms can identify subtler multivariate patterns, the PCA overlap reinforces that salivary ATR-FTIR spectroscopy should not currently be considered a stand-alone diagnostic test.

The biochemical characterization of saliva by FTIR spectroscopy has been consolidated as a promising strategy for the noninvasive diagnosis of several pathologies, including potentially malignant lesions of the oral cavity [17]. The present study reinforces this perspective by demonstrating relevant spectral variations between the groups analyzed, with emphasis on the regions between 900 and 1800 cm⁻¹, associated with components such as proteins (amides I and II), nucleic acids and collagen — biomolecules already reported in the literature as altered in dysplastic or inflammatory processes [18, 19].

The findings of this study, particularly band 1076 cm^-1^, associated with symmetric stretching of phosphate groups in nucleic acids and the skeletal conformation of DNA, support the presence of spectral contributions from genetic material in saliva [20]. The band around 1646 cm-1 may also include contributions from nucleic-acid base vibrations; this region is strongly influenced by the amide I band of proteins and should therefore be interpreted with caution. Previous studies have demonstrated that FTIR spectroscopy can detect structural and conformational changes in nucleic acids and, more recently, subtle alterations associated with DNA methylation status [21].

Classification performance was estimated using participant-level stratified group 10-fold cross-validation, ensuring that replicate spectra from the same individual were never split between training and validation partitions. The best-performing model (Random Forest) achieved an accuracy of 0.740 ± 0.127 and an AUC of 0.845 ± 0.135. These values are lower than those obtained under spectrum-level partitioning, confirming that replicate-level splitting inflates apparent performance in replicate-based spectroscopic designs. The majority-class baseline for this dataset is 0.569, so the models retain discriminative capacity; however, the wide dispersion across folds (per-fold AUC 0.605–1.000) reflects the limited number of participants per validation partition and indicates that these estimates should be regarded as preliminary.

Furthermore, the literature has already validated the use of saliva as a diagnostic biofluid in cases of diabetes, through the identification of biomarkers such as glycated hemoglobin and oxidative enzymes [19, 22]. In the present work, the use of FTIR combined with machine learning algorithms (Extra Trees, Random Forest, XGBoost) obtained high performance (AUC > 0.92; F1-score > 0.86), reinforcing its robustness for diagnostic application.

The relatively small sample size represents an important limitation of the present study. Although the inclusion of 20 patients with oral leukoplakia and 20 healthy individuals allowed an initial investigation of salivary biochemical differences, this cohort may not capture the complete clinical and biological heterogeneity of oral leukoplakia. Moreover, classification metrics obtained from small datasets may be optimistic and particularly sensitive to the composition of the training and testing subsets. Therefore, the reported accuracy and area under the receiver operating characteristic curve should be interpreted as preliminary estimates that require confirmation in larger and independent populations.

This study presents certain limitations that should be considered when interpreting the findings: the relatively small sample size and recruitment within a specific clinical setting may limit the generalizability of the results to broader, more heterogeneous populations and the absence of an independent external validation cohort limits the extent to which the reported classification performance can be generalized to other populations.

This study contributes to the consolidation of FTIR as a promising technique for screening potentially malignant oral lesions. The application of machine learning models on spectral data proved to be effective in detecting salivary biochemical alterations, with the future possibility of large-scale clinical application for potential auxiliary approach for the characterization of oral leukoplakia. Future studies should expand the case series, integrate complementary molecular analyses and explore the potential of FTIR in risk stratification of patients with oral lesions or systemic conditions, contributing to advances in both dentistry and preventive medicine.

The combination of salivary ATR-FTIR spectroscopy and machine learning identified biochemical patterns associated with oral leukoplakia and demonstrated promising preliminary classification performance. However, these findings represent proof-of-concept evidence and do not establish salivary FTIR spectroscopy as an optical biopsy or a stand-alone diagnostic test. Larger prospective cohorts, standardized collection and analytical procedures, independent external validation, and complementary molecular investigations are required to determine the reproducibility and clinical utility of this approach. If subsequently validated, salivary ATR-FTIR spectroscopy may contribute as a rapid and minimally invasive adjunctive tool for screening or risk stratification, while clinical examination and histopathological analysis remain essential for definitive diagnosis.

## Conclusions

The potential of FTIR spectroscopy applied to saliva as a promising tool for the early diagnosis of potentially malignant oral lesions was described. Detection of biochemical differences between the analyzed groups, especially in spectral regions associated with proteins, nucleic acids, and collagen, highlights the sensitivity of the technique to metabolic and cellular changes related to dysplastic processes.

Future investigations should include larger, prospectively recruited, and demographically diverse cohorts, preferably through multicenter designs and independent external validation. Additionally, the integration of FTIR spectroscopy with proteomic, metabolomic, transcriptomic, or targeted biochemical analyses could help identify the molecular contributors to the discriminative spectral regions. Because FTIR spectroscopy provides a global biochemical fingerprint, spectral bands associated with proteins, nucleic acids, and collagen should be interpreted as representing broad molecular classes rather than specific biomarkers. Orthogonal molecular validation may therefore improve the biological interpretation of the spectral findings and clarify their relationship with epithelial dysplasia, disease progression, and malignant transformation risk.

We also demonstrated the integration with machine learning models with high accuracy and discriminative power, establishing a robust and non-invasive approach for clinical use. Despite limitations such as sample size and the need for standardized protocols, the findings of this study pave the way for the development of faster, safer, and more accessible diagnostics. Saliva as a diagnostic biofluid, combined with spectroscopic technologies, may transform clinical practice by improving the effectiveness of screening and monitoring patients at risk for oral cancer.

## Data Availability

All data supporting the results presented in this study are described and available within the manuscript and/or its supplementary materials. Additional information, clarifications regarding the methodology employed, or data necessary for understanding and reproducing the research can be provided by the authors upon request. The authors remain available to address any questions related to the study.
